# Dietary Total Antioxidant Capacity Is Inversely Associated with Prediabetes and Insulin Resistance in Bialystok PLUS Population

**DOI:** 10.3390/antiox11020283

**Published:** 2022-01-29

**Authors:** Monika Cyuńczyk, Małgorzata Elżbieta Zujko, Jacek Jamiołkowski, Kinga Zujko, Magda Łapińska, Magdalena Zalewska, Marcin Kondraciuk, Anna Maria Witkowska, Karol Adam Kamiński

**Affiliations:** 1 Department of Food Biotechnology, Faculty of Health Sciences, Medical University of Bialystok, Szpitalna 37, 15-295 Bialystok, Poland; monika.cyunczyk@umb.edu.pl (M.C.); z_kinga@wp.pl (K.Z.); witam@umb.edu.pl (A.M.W.); 2 Department of Population Medicine and Lifestyle Diseases Prevention, Medical University of Bialystok, Waszyngtona 13a, 15-269 Bialystok, Poland; jacek.jamiolkowski@umb.edu.pl (J.J.); magda.lapinska@umb.edu.pl (M.Ł.); magdaz@umb.edu.pl (M.Z.); marcin.kondraciuk@umb.edu.pl (M.K.); karol.kaminski@umb.edu.pl (K.A.K.)

**Keywords:** diabetes, prediabetes, HOMA-IR, dietary total antioxidant capacity, FRAP, population

## Abstract

The aim of this study was to assess the relationship between the dietary total antioxidant capacity (DTAC) and occurrence of prediabetes, diabetes and insulin resistance in the Bialystok PLUS (Polish Longitudinal University Study) population. Daily food consumption was estimated by 3-days 24-h dietary recalls. DTAC was calculated using the date of food consumption and antioxidant potential of foods measured by FRAP (ferric ion reducing antioxidant potential) method. The following measurements were performed to identify prediabetes, diabetes and HOMA-IR: fasting glucose (FG), 2h postprandial glucose level (2h-PG), fasting insulin (FI), glycated hemoglobin HbA1c. Logistic regression models were used to assess the relationship between DTAC and prediabetes and diabetes. This study demonstrated that higher quartile of DTAC, after adjustment for confounding variables, was significantly associated with a reduced odds ratio for the prevalence of prediabetes in Bialystok PLUS population aged 35–65 years. DTAC was also significantly inversely associated with HOMA-IR in multivariate linear regression model. DTAC was positively related to individual dietary antioxidants (polyphenols, antioxidant vitamins and minerals). Reduced DTAC may be considered as an additional risk factor for the development of diabetes. Therefore, dietary recommendations for prevention and therapy of diabetes should take into account the high DTAC.

## 1. Introduction

Diabetes mellitus is a major public health problem. Due to its increasing incidence, it has been considered as a non-communicable pandemic of the 21st century. Globally, 463 million adults (20–79 years) had diabetes in 2019 with 90% of them being type 2 diabetes mellitus (T2DM) cases, and this number is projected to reach 578 million by 2030 and 700 million by 2045 (51% increase from 2019 to 2045). Furthermore, 50% of adults with diabetes are undiagnosed. In Poland diabetes affects 8.1% of the population [1]. The occurrence of T2DM in Poland is particularly high among patients with coronary artery disease [2].

The incidence of chronic diseases is related to five modifiable lifestyle risk factors, such as unhealthy diet, extensive alcohol consumption, overweight and obesity, cigarette smoking and physical inactivity. The PREDIMED study for the first time indicated that the Mediterranean diet, rich in vitamins, polyphenols, fibers, mono- and polyunsaturated fatty acids, probiotics, and low glycemic foods, reduced the risk of developing T2DM [3]. However, studies carried out in the following years showed that trend in nutrient intake in Mediterranean countries has changed over the years, progressively abandoning the traditional reference Mediterranean type [4]. This suggests the necessity to assess the dietary patterns and nutrients important in reducing T2DM in rapidly changing societies.

In the pathogenesis of T2DM an important role plays oxidative stress defined as an imbalance between generation of prooxidants (reactive oxygen—ROS, nitrogen—RNS and chlorine species—RCS) and the body antioxidant system (enzymatic: superoxide dismutase, glutathione peroxidase and catalase, and non-enzymatic: metal binding proteins, glutathione, uric acid, melatonin, bilirubin and polyamines). The overproduction of free radicals in chronic hyperglycemia is associated with non-enzymatic protein glycation, glucose oxidation and lipid peroxidation. Oxidative stress can impair insulin signaling pathways and induce cytotoxicity in pancreatic β-cells, leading to insulin resistance and diabetes [5,6,7].

Exogenous antioxidants, such as polyphenols (flavonoids, phenolic acids, stilbenes and lignans), vitamin C, E, carotenoids and minerals (Cu, Mn, Zn, Se, Fe), can support the action of endogenous antioxidants in alleviating the destructive effects of oxidative stress. Polyphenols may reduce the risk of T2DM by lowering postprandial glucose level, modulating glucose transport, affecting insulin signaling pathways, and by protecting against pancreatic β-cell damage. Vitamins A, C and carotenoids directly or indirectly scavenge free radicals and exert stimulating effects on endogenous antioxidant and immunomodulatory functions. Minerals are cofactors for antioxidant enzymes, which are the first line of defense against free radicals [8,9,10].

Increasing evidence from population studies suggests that individual dietary antioxidants, such as vitamin C [11], vitamin E [12], carotenoids [13] and polyphenols [14,15] may reduce the risk of T2DM in different population. However, the whole diet contains various antioxidants with additional or synergistic effects. Previous case-control study showed that diet of T2DM patients was poor in antioxidants (total antioxidant capacity, total polyphenols, flavonoids, vitamin C), despite increased demand (higher serum oxidative stress markers), especially in patients with long-lasting disease [16]. In the other intervention study individual diet modification in terms of higher antioxidants intake (total polyphenols, flavonoids and anthocyanins), reduced the hyperglycemia in patients with metabolic syndrome [17].

The epidemiological evidence of a relationship between DTAC and T2DM is quite limited. Only six cohort, cross-sectional and case-control studies showed that DTAC was inversely associated with the risk of T2DM [18,19], as well as insulin resistance [19,20], impaired glucose tolerance [21], prediabetes [22] and gestational diabetes [23]. However, these associations were found only for women [18,21], pregnant women [23], older people [19] and older people with a cardiometabolic risk profile [20]. Only one study was conducted in a middle-aged population (35–65 years), but only in people with prediabetes [22].

The aim of this study was to assess the relationship between the dietary total antioxidant capacity and prevalence of prediabetes, diabetes and insulin resistance in Bialystok PLUS (Polish Longitudinal University Study) middle-aged population (35–65 years) of both sexes. This is the first cross-sectional study to examine these associations in Polish population, supplementing the existing knowledge in this field.

## 2. Materials and Methods

### 2.1. Ethical Approval

The study was conducted in accordance with the Helsinki Declaration and Good Clinical Practice, as well as was approved by the Ethics Committee of the Medical University of Bialystok, Poland (approval numbers: R-I-002/108/2016, date of approval 31 March 2016 and R-I-002/192/2019, date of approval 28 March 2019). Informed consent was given by all participants of the study.

### 2.2. Study Population

Subjects were participants of the Bialystok PLUS study (Polish Longitudinal University Study) performed from July 2017 to February 2020, which is a cross-sectional study aimed at investigating the determinants of chronic non-communicable diseases in a random sample of Bialystok residents aged 20–80 years [24]. From the Bialystok adult population, a sample of 3195 individuals was drawn from the Department of State Registry database, the Ministry of Internal Affairs (PESEL register). Subsequently, 1729 persons did not consent to participate in this study or were unavailable. Therefore, the initial study group consisted of 1466 subjects (a response rate of 45.9%). After taking into account the exclusion criteria: age < 35 and >65 years, cancer and cardiovascular events (stroke, heart failure, myocardial infarction) in interviews and incomplete nutritional questionnaires, the daily energy intake < 500 kcal or >5000 kcal, 413 participants were qualified for the study. The data were collected through standardized health examinations in specially-equipped examination centers.

### 2.3. Dietary Assesment

Dietary assessment was performed by a trained interviewer, using 3-days 24-h dietary recalls (two randomly selected weekdays and one weekend day). Food portion sizes were estimated using an album with photographs of the most consumed in Poland food products and dishes [25]. Energy and nutrients from dietary assessments were calculated using the Diet 6.0 computer program (developed by the National Institute of Public Health, Poland).

### 2.4. Estimation of DTAC and DTPI

DTAC (dietary total antioxidant capacity) was determined by multiplying the daily consumption of individual food items by antioxidant potential of these foods. Antioxidant potential of foods, measured by FRAP method (ferric ion reducing antioxidant potential), was obtained from the Antioxidant Food Table published by Carlsen, who determined the antioxidant content of over 3100 types of food and beverages [26]. Some missing values have been supplemented with our database [27,28].

DTPI (dietary total polyphenol intake) was estimated using the online Phenol-Explorer database [29] and our database [27,28].

### 2.5. Ascertainment of Prediabetes, Diabetes and HOMA-IR

Peripheral intravenous blood samples were collected in the examination center in the morning after at least 8 h of fasting. Next, the samples were immediately centrifuged and stored at −70 °C until analysis. Glucose level was performed in serum using enzymatic-colorimetric method on the analyzer Cobas c111 (ROCHE, Meylan, Isère, France). Insulin level was measured by the electrochemiluminescence ECLIA method, using Cobas e411 analyzer (ROCHE, Meylan, Isère, France). Glycated hemoglobin HbA1c was determined by high-performance liquid chromatography HPLC method on the D-10 Hemoglobin Testing System Bio-Rad analyzer (Bio-Rad, Hercules, CA, USA).

In accordance with American Diabetes Association [30] and Polish Diabetes Society [31] recommendations, we defined type 2 diabetes as a fasting glucose level ≥126 mg/dL (7.0 mmol/L) or 2-h postprandial glucose ≥200 mg/dL (11.1 mmol/L) during oral glucose tolerance test, or glycated hemoglobin HbA1c ≥ 6.5% (48 mmol/mol), or participants were taking medication. We defined prediabetes as a fasting glucose level ≥100 mg/dL (5.6 mmol/L) and ≤125 mg/dL (6.9 mmol/L) or 2-h postprandial glucose ≥140 mg/dL (7.8 mmol/L) and ≤199 mg/dL (11.0 mmol/L) during oral glucose tolerance test, or glycated hemoglobin HbA1c ≥ 5.7% (39 mmol/mol) and ≤6.4% (47 mmol/mol). HOMA-IR index was calculated using the following formula: HOMA-IR = [fasting serum insulin (mU/L) × fasting plasma glucose (mg/dL)]/405 [32].

### 2.6. Analysis of Confounding Variables

The following potentially confounding variables were included in our analyses: age, sex, family history of diabetes, level of education, physical activity, dyslipidemia, hypertension, body mass index, waist circumference, smoking status, daily alcohol intake and daily energy intake.

The following information: age, sex, family history of diabetes and level of education were collected from self-reported questionnaires, which were designed for the Bialystok PLUS study. Physical activity status was evaluated using International Physical Activity Questionnaires (IPAQ) [33]. Smoking status was measured using exhaled carbon monoxide test with Micro+ Smokerlyzer™ device (Bedfont Scientific Ltd., Harrietsham, UK).

Dyslipidemia was defined as elevated total cholesterol (T-Chol ≥ 190 mg/dL) level or elevated low-density lipoprotein cholesterol (LDL-Chol ≥ 115 mg/dL) level, or low levels of high-density lipoprotein cholesterol (HDL-Chol < 40 mg/dL for men and <48 mg/dL for women), or elevated triglycerides (TG ≥ 150 mg/dL) level, or participants were taking medication [34]. T-Chol, LDL-Chol, HDL-Chol and TG were performed in serum using enzymatic-colorimetric method on the analyzer Cobas c111 (ROCHE, Meylan, Isère, France).

Hypertension was defined as systolic blood pressure (BPs) ≥140 mm Hg and/or diastolic blood pressure (BPd) ≥ 90 mm Hg, or participants were taking medication [35]. Blood pressure was measured using automatic Omron M6 Comfort device (Omron Healthcare, Kyoto, Japan) on the right arm in the sitting position after 5 min of rest.

Anthropometric measurements including body mass, height and waist circumference were performed using standardized procedures. Body mass index (BMI) was calculated as body mass in kilograms divided by squared height in meters (kg/m^2^) [36].

Daily energy and alcohol intakes were calculated from data obtained from 3-days 24-h dietary recalls.

### 2.7. Statistical Analysis

The normality of distributions of continuous variables was tested using Shapiro–Wilk test. Due to significant deviation of some variables from the normal distribution, nonparametric methods were used in statistical analyses. Comparisons between multiple continuous variables were conducted using Kruskal–Wallis test. Differences between groups for categorical data were detected using Pearson’s chi-squared test for linear trend. To quantify relationships between continuous variables nonparametric Spearman correlations were performed. Logistic regression models were used to assess the relationship between DTAC and prevalence of prediabetes and diabetes. Three models were performed: 1 model—crude data; 2 model—data adjusted for age, sex and daily energy intake; 3 model—data multivariable-adjusted (age, sex, family history of diabetes, educational level, smoking status, physical activity, dyslipidemia, hypertension, BMI, waist circumference, alcohol consumption, daily energy intake). Results were presented as odds ratios (ORs) and 95% confidence intervals (CIs) of the associations between quartiles (Q1–Q4) of DTAC and prediabetes and diabetes. Linear regression was used to investigate the relationship between DTAC and HOMA-IR (one crude and two adjusted models). Results were presented as regression coefficient (β) and 95% confidence interval (CI). All statistical hypotheses were verified at 0.05. Statistical calculations were performed with IBM SPSS Statistics 27.0 software [37,38].

## 3. Results

In this study, among 413 analyzed participants, normoglycemia was detected for 171 (41.40%) subjects, while 202 (48.91%) persons had prediabetes and 40 persons (9.69%) diabetes. All prediabetes and 55% diabetes participants were undiagnosed. The mean age was 49.84 ± 9.47 and 40% were male. Over 60% of participants were overweight or obese. The mean dietary total antioxidant capacity in Bialystok PLUS population was 12.417 ± 5.437 (range 1.511–43.212) mmol/day.

The baseline characteristics of the study population by quartiles of dietary total antioxidant capacity are shown in Table 1.

Compared with lower quartiles, a higher percentage of individuals in the higher quartiles of DTAC had higher educational level (*p* = 0.016) and lower smoking status (*p* = 0.041). Moreover, participants in the higher quartiles of DTAC had lower BMI (*p* = 0.043) and higher daily energy intake (*p* = 0.001). No significant differences were found regarding age, sex, family history of diabetes, physical activity, dyslipidemia, hypertension, waist circumference, as well as alcohol consumption.

The odds ratios for prediabetes and diabetes by quartiles of DTAC were evaluated using multiple logistic regression analysis (Table 2). Three models were tested: model 1—crude data; model 2—data adjusted for age, sex and daily energy intake; model 3—data adjusted for age, sex, family history of diabetes, educational level, smoking status, physical activity, dyslipidemia, hypertension, BMI, waist circumference, alcohol consumption, daily energy intake. The first quartile (Q1) in each model was adopted as a reference. It was found that higher quartile of DTAC, crude (model 1) and after adjustment for confounding variables (model 2 and model 3) was significantly associated with a reduced odds ratio for the prevalence of prediabetes (FG = 100–125 mg/dL). In model 3—Q2 vs. Q1: OR (95% CI) = 0.482 (0.263–0.884), Q3 vs. Q1: OR (95% CI) = 0.583 (0.309–0.945). No significant association was found in model 2 and model 3 for Q4 vs. Q1.

Table 3 presents the results of linear regression analysis of DTAC and HOMA-IR. The univariate (model 1) and multivariate analysis (model 2 and 3) showed a significant association between DTAC and HOMA-IR. In model 3 (adjusted for age, sex, family history of diabetes, educational level, smoking status, physical activity, dyslipidemia, hypertension, BMI, waist circumference, alcohol consumption, daily energy intake) DTAC was inversely associated with HOMA-IR (β = −0.39, CI = −0.75–−0.14, *p* = 0.024, R^2^ = 0.07). DTAC explained 7% of the variation in HOMA-IR.

In Table 4 are presented correlations between DTAP and individual dietary antioxidants. A significant, positive, strong correlation was shown between DTAC and DTPI (r = 0.867, *p* < 0.001); moderate correlations between DTAC and iron (r = 0.442, *p* < 0.001), copper (r = 0.468, *p* < 0.001) and manganese (r = 0.431, *p* < 0.001); weak correlations between DTAC and vitamin C (r = 0.363, *p* = 0.011), vitamin E (r = 0.348, *p* = 0.008) and zinc (r = 0.321, *p* = 0.009); and a weak correlation between DTAC and β-carotene (r = 0.182, *p* = 0.012).

The main food sources of DTAC in the studied population were given in Table 5. It was shown that plant foods, such as coffee infusion (33.8%), fruits and juices (16.7%), tea infusion (12.2%), nuts and seeds (8.6%), and vegetables without potatoes (8.2%) represented the major sources of DTAC (79.5%). Moreover, important contributors of DTAC were potatoes (3.2%), wheat and wholegrain cereal products (3.8%), chocolates and cacao (3.6%) and alcohol (2.3%).

## 4. Discussion

To our knowledge, this is the first study to examine relationship between dietary total antioxidant capacity and occurrence of prediabetes, diabetes and insulin resistance in Polish population in cross-sectional study. In this study we analyzed Bialystok PLUS middle-aged (35–65 years) population of both sexes. It was shown that in the studied group over 9% participants had diabetes, which is in agreement with occurrence of diabetes among the whole Polish population (over 8%) [1]. However, it is very disturbing that almost 49% of subjects had prediabetes. Moreover, all prediabetes and more than half diabetes were undiagnosed. Especially worrying is the fact that one third of the studied population has a family history of diabetes.

It is well known that modification of lifestyle, including healthy nutrition, regular physical activity of moderate intensity, maintaining a normal body weight, and avoiding tobacco use and excessive alcohol consumption is the primary approach for type 2 diabetes mellitus prevention and therapy [39,40]. Visceral obesity is associated with an increased risk of metabolic syndrome (MetS) and nonalcoholic fatty liver disease (NAFLD). In NAFLD an increase in hepatic lipogenesis and a lack of suppression of the lipolysis in the adipose tissue is observed, which determines an increase in the fatty acids flow inside the liver and leads to insulin resistance. Insulin resistance is the most important factor in the development of prediabetes and T2DM [41].

In this study over 60% of participants were overweight or obese, over 20% smoked cigarettes and about 70% had low physical activity. The consumption of alcohol in the study group was relatively low. In the WOBASZ study (Multi-centre National Population Health Examination Survey), performed to represent the Polish population, ethanol consumption (mainly vodka and beer) was less than 15 g/day in 80% Polish adults, but 13% consumed 15–30 g/day and 7% over 30 g/day [42]. Moreover, 30% of Polish men and 21% of Polish women smoked, but the percentage of smokers decreased over the years [43]. The prevalence of overweight and obesity in Poland was at the level of 67% in men and 56% in women and an upward trend was observed [44].

In this study daily energy consumption was relatively low, which could be related to low alcohol consumption. A previous study conducted in a Mediterranean country (Italy) showed that energy intake and alcohol consumption decreased over three decades, but the trend in valuable nutrient intake has worsened [4]. It is worth noting that in this study individuals in the higher quartiles of DTAC had higher educational level and daily energy intake, but lower BMI and smoking status. It can be assumed that people with higher education led a healthier lifestyle and chose food products with higher nutritional value, which is in accordance with the previous study [45].

Currently, much attention is paid to the study of the relationship between eating patterns and the development of diabetes. A systematic review of randomized clinical trials supports the view that vegan, vegetarian and Mediterranean dietary patterns should be implemented to stop the diabetes pandemic [46]. All healthy eating patterns, based on fruits and vegetables, are sources of antioxidants. Although many studies highlight the important role of individual dietary antioxidants (polyphenols, antioxidant vitamins and minerals) in preventing diabetes [11,12,13,14,15], investigating the antioxidant capacity of the whole diet seems more appropriate. In this study DTAC was significantly positively associated with individual dietary antioxidants, such as polyphenols, vitamin C, E, β-carotene, iron, zinc, copper and manganese.

Some authors suggest that DTAC may be a potential marker of diet quality in healthy subjects [47]. The present study showed that participants (both men and women) with higher DTAC were less likely to have prediabetes. Moreover, a significant inverse association was found between DTAC and HOMA-IR.

Our findings are in agreement with previous studies which have investigated relationships between DTAC and diabetes in different populations. In a prospective cohort E3N-EPIC study DTAC was inversely associated with the risk of T2DM, but only in middle-aged French women [18]. In a population-based cohort the Rotterdam Study DTAC was related to a lower risk of T2DM, but not a risk of prediabetes, and was inversely associated with insulin resistance in the Netherlands older subjects (45 years and older) of both sexes [19]. In cross-sectional FLiO study higher DTAC was associated with lower insulin resistance in overweight or obese Spanish adults aged 40–80 years [20]. In the HCS cohort study higher DTAC was associated with better glucose tolerance, particularly among UK overweight and obesity women [21]. In case-control Iranian study higher DTAC was inversely related to prediabetes [22]. In another Iranian hospital-based case-control study, DTAC was associated with lower risk of gestational diabetes mellitus in pregnant women [23]. In a Polish cross-sectional WOBASZ II study, DTAC was inversely related to some metabolic syndrome risk factors, including elevated glucose level in women aged 20 years or older [48]. Our study was conducted in a middle-aged population of both sexes and investigated relationships between DTAC and prediabetes, diabetes and HOMA-IR.

In this study the mean DTAC was 12.417 ± 5.437 mmol/day and ranged widely from 1.511 to 43.212 mmol/day. Our results are in line with a previously published study conducted on a representative sample of the Polish adult population where mean DTAC was 12.313 ± 7.371 mmol/day (range 0.471–191.822) [48]. Mancini and colleagues [18] showed inverse association between DTAC and risk of T2DM, but only to FRAP value about 15 mmol/day. Similarly, in our study, in model 3 we found a significant association between DTAC and prediabetes, but only between Q2 (8.38–11.27 mmol/day) and Q3 (11.2–14.50 mmol/day) vs. Q1 (≤8.37 mmol/day), and not between Q4 (≥14.51 mmol/day) vs. Q1 (≤8.37 mmol/day).

The types of foods that contribute the most to the DTAC vary widely between countries. The main contributors to the DTAC in Polish population with cardiovascular disease were beverages (tea and coffee), vegetables (potatoes, cabbage, beetroots, tomatoes), fruits (apples, strawberries, plums, bananas, oranges, mandarins) and cereal products [49]. In Norwegian women there was coffee, tea, red wine, blueberries, walnuts, oranges, cinnamon and broccoli [50]. In the US population: tea, fruits, fruit juices and dietary supplements [51]. In French women: fruit, vegetables, alcoholic beverages and hot beverages such as tea, chicory and hot chocolate, but in this study, coffee was excluded from the analysis [18]. While in the Netherlands population there was: coffee, fruit, vegetables, tea and chocolate [19]. The principal foods in UK women were coffee, tea, fruit and vegetables [21]. In our study the main food sources of DTAC in Bialystok PLUS population were coffee infusion, fruits and juices, tea infusion, nuts and seeds and vegetables without potatoes (79.5% of DTAC).

Coffee is the main contributor of DTAC in many countries [18,19,21,49,50]. The antioxidant activity of coffee is related to the content of several antioxidants (e.g., chlorogenic, ferulic, caffeic, and n-coumaric acids, lignans, flavonoids, caffeine, trigonelline, melanoidins and minerals) that may attenuate enzymatic or mitochondrial production of ROS. A previous study confirmed the beneficial effect of coffee on diabetes prevention, for drinking of approximately one to two cups on day [52]. Other studies found that habitual coffee drinking helped maintain normal glucose tolerance and improved insulin sensitivity [53]. In our study coffee intake captured almost 34% of DTAC in middle-aged participants of both sexes, in Norwegian women–54% of DTAC [50], while in the Netherlands subjects aged 45 years and older–49% of DTAC [19]. Van der Schaft and colleagues [19] and Mancini and colleagues [18] reported that association between DTAC and diabetes decreased when the contribution of coffee was excluded from the analysis, suggesting that part of this association is explained by coffee intake. However, our previous study conducted in the Polish population with cardiovascular disease showed that tea consumption was 2-fold higher than coffee [49]. Low intake of coffee was also observed in the Mediterranean population [54].

Except coffee, the most important contributors of DTAC in our study were fruit and juices, tea, nuts and seeds, and vegetables without potatoes. Previously, it was shown that higher consumption of fruit and vegetables [55], as well as tea [56] and peanut butter [57] were associated with a lower risk of T2DM.

In summary, a higher dietary antioxidant intake in lower BMI, avoiding smoking and excessive alcohol consumption can prevent diabetes and improve the quality and life expectancy of diabetics, despite relatively low physical activity [58].

The present study also has some strengths and limitations. The strength of this study is that it was a cross-sectional study conducted on a random sample of Bialystok adult residents and we used standardized methods. In addition, the study took into account middle-aged adults (35–65 years), which is the most appropriate for determining the relationship between lifestyle and the development of metabolic diseases. Next, we used 3-day 24-h dietary recalls to collect the data on nutrition. The main limitation of this study is that the dietary antioxidant database is not complete and does not include all food products and supplements. Moreover, the quality of the nutritional interview depends on the memory of the respondents and the accuracy of the recording.

## 5. Conclusions

This study demonstrated that higher quartile of DTAC, after adjustment for confounding variables, was significantly associated with a reduced odds ratio for the prevalence of prediabetes in the Bialystok PLUS population aged 35–65 years. DTAC was also significantly inversely associated with HOMA-IR and positively related to individual dietary antioxidants (polyphenols, antioxidant vitamins and minerals). Reduced DTAC may be considered as an additional risk factor for the development of diabetes. Therefore, dietary recommendations for prevention and therapy of diabetes should take into account the high DTAC.

## Figures and Tables

**Table 1:** Baseline characteristics of the study population by quartiles of dietary total antioxidant capacity (*n* = 413).

Variables	Quartiles of Dietary Total Antioxidant Capacity (mmol/Day)				*p*
	Q1 (≤8.37) *n* = 104	Q2 (8.38–11.27) *n* = 103	Q3 (11.2–14.50) *n* = 103	Q4 (≥14.51) *n* = 103	
Age (years), mean ± SD	49.12 ± 9.43	50.81 ± 9.54	50.13 ± 9.62	49.34 ± 9.41	0.595
Sex, n (%)					
Male	46 (44.23)	44 (42.72)	41 (39.81)	35 (33.98)	0.119
Female	58 (55.77)	59 (57.28)	62 (60.19)	68 (66.02)	
Family history of diabetes, n (%)					
No	67 (64.43)	67 (65.05)	64 (62.14)	68 (66.02)	0.821
Yes	37 (35.57)	36 (34.96)	39 (37.86)	35 (34.98)	
Educational level, n (%)					
Bellow middle	25 (24.04)	18 (17.48)	14 (13.59)	11 (10.68)	0.016
Middle	27 (25.96)	38 (36.89)	32 (31.07)	32 (31.07)	
Higher	52 (50.00)	47 (45.63)	57 (55.34)	60 (58.25)	
Smoking status, n (%)					
No	76 (73.08)	81 (78.64)	85 (82.53)	87 (84.47)	0.041
Yes	28 (26.92)	22 (21.36)	18 (17.47)	16 (15.53)	
Physical activity, n (%)					
Low	75 (72.12)	74 (71.85)	64 (62.14)	65 (63.11)	0.075
Medium	29 (27.88)	29 (28.15)	39 (37.86)	38 (36.89)	
High	0 (0.0)	0 (0.0)	0 (0.0)	0 (0.0)	
Dyslipidemia, *n* (%)	68 (65.39)	62 (60.19)	71 (68.93)	65 (63.11)	0.345
Hypertension, *n* (%)	24 (23.08)	20 (19.42)	25 (24.27)	23 (22.33)	0.312
BMI (kg/m^2^), mean ± SD	27.92 ± 5.13	27.31 ± 4.74	26.63 ±4.61	26.33 ± 5.44	0.043
WC (cm), mean ± SD	88.81 ± 14.32	88.75 ± 13.23	87.12 ± 13.11	85.94 ± 13.75	0.191
Alcohol consumption (g/day) ^1^	2.74 (0.5, 6.6)	3.21 (0.7, 9.3)	5.82 (0.6, 16.3)	5.01 (0.3, 10.7)	0.358
Energy (kcal/day), mean ± SD	1771.1 ± 514.5	1824.2 ± 588.6	2031.5 ± 607.4	2107.1 ± 567.3	0.001

Date are presented as means and standard deviations (SD) for continuous variables, and count (*n*) and percentage (%) for categorical variables; ^1^—Variables are presented as median (interquartile range) due to not normal distribution; Kruskal-Wallis test was used for comparison of continuous variables and chi-square test for linear trend for categorical variables; BMI—body mass index, WC—waist circumference.

**Table 2:** OR (95% CI) for prediabetes and diabetes by quartiles of dietary total antioxidant capacity.

Variables	Quartiles of Dietary Total Antioxidant Capacity (mmol/Day)			
	Q1 (Ref.) (≤8.37)	Q2 (8.38–11.27)	Q3 (11.2–14.50)	Q4 (≥14.51)
FG = 100–125 mg/dL				
Crude OR (95% CI)	1	0.525 (0.302–0.913) *	0.591 (0.340–0.915) *	0.595 (0.342–0.975) *
Adjusted OR (95% CI) ^1^	1	0.397 (0.214–0.735) *	0.469 (0.247–0.890) *	0.526 (0.273–1.003)
Adjusted OR (95% CI) ^2^	1	0.482 (0.263–0.884) *	0.583 (0.309–0.945) *	0.580 (0.303–1.007)
FG ≥ 126 mg/dL				
Crude OR (95% CI)	1	0.867 (0.407–1.345)	0.416 (0.104–1.654)	0.274 (0.056–1.354)
Adjusted OR (95% CI) ^1^	1	0.841 (0.365–1.566)	0.426 (0.098–1.853)	0.317 (0.058–1.741)
Adjusted OR (95% CI) ^2^	1	0.875 (0.465–1.683)	0.512 (0.112–1.337)	0.237 (0.037–1.516)
2h-G = 140–199 mg/dL				
Crude OR (95% CI)	1	0.643 (0.339–1.017)	0.601 (0.363–1.015)	0.653 (0.392–1.018)
Adjusted OR (95% CI) ^1^	1	0.529 (0.368–1.042)	0.597 (0.293–1.017)	0.672 (0.328–1.077)
Adjusted OR (95% CI) ^2^	1	0.738 (0.511–1.105)	0.730 (0.440–1.167)	0.730 (0.482–1.202)
2h-G ≥ 200 mg/dL				
Crude OR (95% CI)	1	0.855 (0.267–1.316)	0.715 (0.286–1.350)	0.454 (0.167–1.471)
Adjusted OR (95% CI) ^1^	1	0.811 (0.234–1.452)	0.721 (0.269–1.081)	0.505 (0.169–1.374)
Adjusted OR (95% CI) ^2^	1	0.966 (0.287–1.559)	0.630 (0.236–1.519)	0.523 (0.174–1.406)
HbA1C = 5.7–6.4%				
Crude OR (95% CI)	1	0.841 (0.598–1.413)	0.959 (0.607–1.645)	0.960 (0.550–1.476)
Adjusted OR (95% CI) ^1^	1	0.899 (0.484–1.572)	0.991 (0.519–1.694)	0.987 (0.507–1.519)
Adjusted OR (95% CI) ^2^	1	0.977 (0.635–1.581)	0.927 (0.742–1.542)	0.965 (0.748–1.568)
HbA1C ≥ 6.5%				
Crude OR (95% CI)	1	0.792 (0.207–2.037)	0.194 (0.022–1.691)	0.000 (0.000–0.000)
Adjusted OR (95% CI) ^1^	1	0.834 (0.202–2.444)	0.243 (0.025–2.313)	0.000 (0.000–0.000)
Adjusted OR (95% CI) ^2^	1	1.011 (0.234–2.380)	0.283 (0.028–2.495)	0.000 (0.000–0.000)

Results are presented as OR (odds ratio) and CI (confidence interval); *—*p* < 0.05; ^1^—analysis adjusted for age, sex and daily energy intake, ^2^—analysis adjusted for age, sex, family history of diabetes, educational level, smoking status, physical activity, dyslipidemia, hypertension, BMI, waist circumference, alcohol consumption, daily energy intake; FG—fasting glucose, 2h-G—2-h plasma glucose during oral glucose tolerance test, HbA1c—hemoglobin A1c.

**Table 3:** Association between dietary total antioxidant capacity and HOMA-IR.

Variable	Model 1			Model 2			Model 3		
	β (95% CI)	*p*	R^2^	β (95% CI)	*p*	R^2^	β (95% CI)	*p*	R^2^
HOMA-IR	−0.52 (−0.78–−0.28)	0.012	0.05	−0.47 (−0.75–−0.26)	0.014	0.06	−0.39 (−0.75–−0.14)	0.024	0.07

Results are presented as β (regression coefficient) and 95% CI (confidence interval); Model 1: crude analysis, Model 2: adjusted for age, sex and daily energy intake, Model 3: adjusted for age, sex, family history of diabetes, educational level, smoking status, physical activity, dyslipidemia, hypertension, BMI, waist circumference, alcohol consumption, daily energy intake.

**Table 4:** Correlation between dietary total antioxidant capacity and individual antioxidants intake.

Variable	Dietary Total Antioxidant Capacity (mmol/Day)	
	r	*p*
Dietary Total Polyphenol Intake (mg/day)	0.867	<0.001
Vitamin C (mg/day)	0.363	0.011
β-carotene (μg/day)	0.182	0.012
Vitamin E (mg/day)	0.348	0.008
Iron (mg)	0.442	<0.001
Zinc (mg)	0.321	0.009
Copper (mg)	0.468	<0.001
Manganese (mg)	0.431	<0.001

r—Spearman correlation coefficient.

**Table 5:** Food contributors to the dietary total antioxidant capacity in Bialystok PLUS population.

Food Contribution	% Contribution to DTAC
Fruits and juices (mainly: apples, bananas, mandarin, oranges)	16.7
Vegetables without potatoes (mainly: tomatoes, pepper, cucumber)	8.2
Potatoes	3.2
Legumes (mainly: soybeans, beans, peas)	0.5
Milk and milk products	0.6
Meat and meat products	0.8
Fish, fish products and sea fruits	0.1
Wheat cereal products (mainly: bread, rolls, pasta)	2.3
Wholegrain cereal products (mainly: bread, pasta, groats)	1.5
Tea infusion (mainly: black tea and green tea)	12.2
Coffee infusion (ground coffee and instant coffee)	33.8
Nuts and seeds (mainly: peanuts, walnuts, hazelnuts)	8.6
Cookies, cakes, sweets	1.1
Chocolates and cacao (mainly: milk and dark chocolate)	3.6
Alcohol (mainly: red wine, bear)	2.3
Oils (mainly: rape oil, sunflower oil)	0.6
Others	3.9

## Data Availability

Data is contained within the article.
